# Phytochemical, Bioactive, and Toxicological Aspects of *Ageratina adenophora* (Spreng.) R.M.King & H.Rob.: A Narrative Review

**DOI:** 10.3390/biom16091276

**Published:** 2026-09-03

**Authors:** Miaomiao Wang, Heng Xu, Zhihui Hao, Haiyang Jiang, Jianzhong Shen, Chongshan Dai

**Affiliations:** 1 State Key Laboratory of Veterinary Public Health and Safety, Department of Veterinary Pharmacology and Toxicology, College of Veterinary Medicine, China Agricultural University, Beijing 100193, China; 2 Technology Innovation Center for Food Safety Surveillance and Detection (Hainan), Sanya Institute of China Agricultural University, Sanya 572025, China

**Keywords:** *A. adenophora*, phytochemistry, biological activity, toxicology, resource utilization

## Abstract

Due to limitations in the use of antibiotics as feed additives to promote growth, the development of safe, effective, and environmentally friendly antibiotic alternatives is a critical and urgent research priority in animal nutrition and veterinary medicine. Plants contain abundant active substances, which are important sources for developing new feed additives or discovering new active substances. *Ageratina adenophora* (Spreng.) R.M.King & H.Rob. (*A. adenophora*) is an herb native to Central America and a globally invasive species that has spread across Asia, Australia, Africa, and other continents. Studies indicated that it possesses a complex chemical composition, with over 100 constituents identified, including terpenoids, flavonoids, and phenolic acids, which exhibit diverse biological activities, including antibacterial, antioxidant, insecticidal, acaricidal, anti-inflammatory, neuroprotective, antidiabetic, antitumor, and gut health regulation effects. However, it also possesses distinct toxicity, primarily targeting organs such as the intestines, liver, and spleen, causing tissue damage by activating specific signaling pathways. This may limit its utilization as a feed additive or in medical drug development. Therefore, this review systematically summarizes the botanical characteristics, geographic distribution, phytochemical composition, biological activities, and toxicological effects of *A. adenophora*. We also discussed its potential application and current challenges. We hope this review can promote its development and utilization as potential feed additives or medicinal resources.

## 1. Introduction

In modern animal husbandry, feed additives played an indispensable role in enhancing growth performance, preventing diseases, and improving feed conversion efficiency [1]. Historically, antibiotics served as a major class of feed additives owing to the potent antimicrobial activity and significant growth-promoting effects. However, the widespread and even indiscriminate use of antibiotics in feed over several decades has precipitated a severe global health crisis [1]. Among the most pressing issues is the widespread proliferation and intensification of antimicrobial resistance (AMR). The emergence and spread of drug-resistant bacterial strains not only lead to therapeutic failures in veterinary medicine but also threaten human medicine by transferring through the food chain and the environment, severely jeopardizing global public health security [2]. Beyond fostering “superbugs,” the use of in-feed antibiotics presents other significant negative impacts. Drug residues can accumulate in animal-derived food products, raising food safety concerns and potentially disrupting the human intestinal microbiota [3]. Furthermore, the non-targeted effects of antibiotics can disrupt the diversity of the animal’s gut microbiota, impair intestinal barrier function and immune homeostasis, thereby affecting nutrient absorption and utilization [3]. In light of these escalating risks, regulatory agencies worldwide have implemented stringent restrictions, exemplified by the European Union’s 2006 and China’s 2020 bans on the use of antibiotics as growth promoters in animal feed [1,4]. This trend signals the dawn of a post-antibiotic era, making the search for safe, effective, and environmentally friendly antibiotic alternatives a critical and urgent research priority in animal nutrition and veterinary medicine.

Plant extracts are rich in diverse secondary metabolites such as polyphenols, alkaloids, and essential oils [5]. These active ingredients exert multi-targeted biological activities, including antimicrobial, anti-inflammatory, antioxidant, and immunomodulatory effects, through synergistic interactions. More importantly, the natural attributes of plant-derived additives confer excellent ecological compatibility, i.e., their residues are readily biodegradable, minimizing environmental persistence and thus reducing the risk of contaminating the food chain. Studies have shown that specific plant extracts can maintain gut health by modulating the composition of the gut microbial community and enhancing intestinal epithelial barrier function [1]. Therefore, the development of feed additives based on natural products not only aligns with the global advocacy for “green” and sustainable animal farming practices but also represents a crucial strategic direction for combating the AMR crisis and ensuring the safety of animal-derived foods.

*Ageratina adenophora* (Spreng.) R.M.King & H.Rob. (*A. adenophora*) is a perennial invasive herbaceous plant of the Asteraceae family, and is native to Mexico and Costa Rica in Central America. It has now spread widely across multiple continents including Asia, Australia, and Africa [6]. Currently, its invasion covers over 30 million hectares in Southwest China and parts of South China, posing a serious threat to ecosystem stability [7,8,9]. This plant exhibits a complex and diverse chemical compositions, including terpenoids (sesquiterpenes, monoterpenes), flavonoids, phenolics, and cyclodipeptides. Among these, terpenoids, especially sesquiterpenes, constitute the major compound class of *A. adenophora*, and cadinene-type sesquiterpenes are regarded as its characteristic core constituents [10,11]. Based on this complex phytochemical foundation, *A. adenophora* exhibits diverse biological activities, including antimicrobial effects against microorganisms, insecticidal and acaricidal actions [11,12], and significant antioxidant and anti-inflammatory effects [11,13]. Additionally, its metabolites have demonstrated potential antitumor activity [14]. For example, the essential oil of *A. adenophora* displayed bactericidal activity against multiple bacteria in antimicrobial research [15]. Endophytic fungi isolated from *A. adenophora* also showed antibacterial potential against methicillin-resistant *Staphylococcus aureus* [9]. Moreover, dried *A. adenophora* biomass has been evaluated as a bio-fumigant to suppress *Pythium aphanidermatum*, and leaf extracts exhibited antioxidant and anti-inflammatory effects in oxidative-injury models of Caco-2 cells [16]. However, its exploration for applications in feed has been constrained due to concurrent reports of hepatotoxicity and other safety risks. 

This article was designed as a narrative review rather than a formal systematic review. Relevant literature published up to July 2026 was identified through searches of the Web of Science Core Collection, CNKI, and PubMed using combinations of terms related to *Ageratina adenophora*, phytochemistry, biological activity, antimicrobial activity, toxicity, toxicology, detoxification, and feed utilization. Additional publications were identified by screening the reference lists of relevant studies and reviews. Peer-reviewed studies directly addressing the botanical characteristics, geographic distribution, chemical constituents, biological activities, toxicological effects, detoxification strategies, or feed applications of *A. adenophora* were considered, whereas duplicate records, conference abstracts, studies lacking sufficient methodological information, and publications without direct relevance to the scope of this review were excluded. Owing to the substantial heterogeneity in experimental models, preparations, exposure regimens, and outcome measures, no formal risk-of-bias assessment or meta-analysis was conducted. Instead, the evidence was synthesized qualitatively, with greater emphasis placed on studies providing clearly defined experimental conditions, quantitative activity or toxicity data, and mechanistic validation. On this basis, the present review critically integrates current knowledge of the phytochemistry, bioactivities, toxicological risks, detoxification approaches, and potential feed applications of *A. adenophora*, with the aim of identifying both its resource value and the evidence gaps that must be addressed before practical utilization.

## 2. Botanical Characteristics and Geographic Distribution

*A. adenophora* is classified within the genus *Ageratina* within the Asteraceae family. It is a perennial herb or subshrub with a chromosome number of 2*n* = 51. It is also called *Eupatorium adenophorum* Spreng., and it has several synonyms [17]. Plants attain a height of 1–3 m, with stems that are erect and clustered, and that are dark purple or green, covered in glandular hairs, and bearing opposite, ascending branches [18]. The leaves are categorized as either opposite, triangular or rhombic-ovate in shape, 4–15 cm long, with irregularly serrated margins, three basal veins, and petioles 1–6 cm long [19]. Capitula densely corymbose, the involucre broadly campanulate, with 3–4 layers of involucre bracts, tubular flowers white or pale purple, achenes pentagonal, apex with white bristly pappus, wind-dispersed [17].

*A. adenophora*, native to Mexico and Costa Rica in Central America, was introduced to Lincang, Yunnan Province, China from Myanmar in the 1940s [20]. It subsequently spread northeastward at an average annual rate of 20 km and is now widely distributed across southwestern and southern Chinese provinces including Yunnan, Guizhou, Sichuan, Guangxi, and Chongqing, with an invasion area exceeding 30 million hectares [19,21]. Globally, it has invaded parts of Asia, Africa, Oceania, and the Americas, including the fog belt of KwaZulu-Natal in South Africa, New South Wales in Australia, and Oahu in the Hawaiian Islands [20]. This species shows a marked preference for fertile, moist, and frequently disturbed habitats, such as stream banks, roadside verges, and forest clearings. It occurs across a broad elevational range of 500–2500 m and is particularly competitive in humid, high-rainfall environments, where it can rapidly establish dense, dominant stands [17,22].

## 3. Phytochemistry

Phytochemical studies have identified over 100 chemical constituents in *A. adenophora* (some of which are shown in Table 1), primarily isolated from the whole plant, leaves, and endophytic fungal tissues [23,24]. Notably, terpenoids (especially sesquiterpenes) constitute the major compound class, with cadinene-type sesquiterpenes representing core defence-related components [25,26]. Another significant group of active components is flavonoids, with the total flavonoid content in some populations of whole plants reaching 12.8 mg/g [24,27]. The total phenolic content of dried plant samples is 98.6 μg/g, notably including higher levels of caffeic acid and p-coumaric acid [20,24]. Among the terpenoids, geraniol and geraniol D have been shown to exhibit antioxidant, antibacterial, and insecticidal activities [9,23,27]. Additionally, amorpha-4,7(11)-diene (a sesquiterpene biosynthetic intermediate) [28], cyclo(*L*-proline-*L*-valine) (a novel cyclodipeptide) [10], and phorbol A (a novel terpene from endophytes) represent novel compounds first isolated from *A. adenophora* and its endophytes [13]. Other constituents include volatile oils, alkaloids, fatty acid esters, sterols, sugars, and other specialized metabolites [9,23,29].

### 3.1. Sesquiterpenes

Sesquiterpenes are representative compounds of *A. adenophora*, featuring a 15-carbon (C_15_) terpenoid backbone. These compounds are classified into several subclasses, including cadinene, and farnesene-type sesquiterpenes. The structural differences among these subclasses significantly influence their biological activities. The insecticidal and antibacterial activities of sesquiterpenes depend on the number and position of oxygen-containing functional groups in their structures, as well as the configuration of double bonds present [32]. For example, the cadinene sesquiterpene (9-oxo-10,11-dehydrodehydropangotenone, also named as euptox A) contains a carbonyl substituent at the 9-position of its skeleton. This structural feature is closely associated with its pronounced insect feeding deterrent activity and toxicity, which may function by disrupting insect energy metabolism [33]. The linear skeleton of the sesquiterpene biosynthetic intermediate (amorpha-4,7(11)-diene) serves as a key precursor for the subsequent formation of oxygen-containing active sesquiterpenes, and its structural transformation directly influences the chemical defense capacity of *A. adenophora* [28]. Liu et al. identified 9β-hydroxyageraphorone, which activates the salicylic acid signaling pathway by upregulating MYB (v-myb avian myeloblastosis viral oncogene homolog) and DOF (DNA binding with One Finger) genes, thereby enhancing tobacco resistance to Tobacco Mosaic Virus [34]. These findings highlight the importance of structure–activity relationships in shaping the biological functions of sesquiterpenes. In *A. adenophora*, sesquiterpenes accumulate predominantly in the leaves, with concentrations substantially higher than those in the roots and stems and peaking during the vigorous summer growth period, potentially reflecting increased defensive demands against insect herbivores [22].

### 3.2. Flavonoids

Flavonoids constitute major bioactive components in *A. adenophora*, featuring a 2-phenylchromenone backbone. These compounds are classified into several subclasses, including flavonoid aglycones and flavonoid glycosides, as well as other related compounds [31].

The structural differences among these subclasses exert a substantial influence on their biological activities. The antioxidant and anti-inflammatory properties of flavonoids depend on the number and position of hydroxyl groups in their structure. Hydroxylation at positions 3, 5, and 7 is crucial for enhancing antioxidant activity, as it neutralizes free radicals by providing electrons [30]. For example, quercetin has hydroxyl substitutions at positions 3, 5, 7, 3′, and 4′ of the flavonoid skeleton. This structural feature endows it with strong DPPH free radical scavenging ability while also exerting anti-inflammatory effects by inhibiting the release of inflammatory factors [9]. Additionally, the phenolic hydroxyl groups of flavonoid molecules bind to the active center of acetylcholinesterase while simultaneously acting on the peripheral site of the enzyme, with inhibition efficiency positively correlated with the number of phenolic hydroxyl groups [31].

### 3.3. Cyclodipeptides

Cyclodipeptides are distinctive bioactive compounds produced by endophytic bacteria within the *A. adenophora*. They feature a fundamental backbone formed by two amino acids cyclized via a peptide bond. They are classified into multiple subclasses, including cyclic (*L*-proline-*L*-valine) types, cyclic (*L*-leucine-*L*-proline) types, and cyclic (*L*-alanine-*L*-proline) types [10].

Cyclic dipeptides containing proline contribute to antibacterial activity, and their efficacy appears to depend on amino acid composition and stereochemical configuration. In the study by Ren et al., cyclo (*L*-Pro-*L*-Val) and cyclo (*L*-Leu-*L*-Pro) showed mild inhibitory activity against *Staphylococcus aureus* and *Escherichia coli* [10]. The cyclic structure of cyclodipeptides confers stability, enabling them to function long-term as signaling molecules or defense compounds in plant-microbe interactions [35].

## 4. Biological Activities of *A. adenophora*

Based on the various bioactive components, such as sesquiterpenes, flavonoids, and cyclic dipeptides, *A. adenophora* exhibited multiple biological activities, including antimicrobial, antioxidant, neuroprotective, antidiabetic, antitumor, and gut health regulation effects. In the following sections, we will discuss them in detail. 

### 4.1. Antibacterial Activity of Extracts

Studies have shown that solvent extracts, essential oils, and endophyte-derived metabolites from *A. adenophora* exhibit notable antibacterial activity (Table 2), characterized by marked species selectivity and clear concentration dependence. For example, Zhang et al. prepared an *A. adenophora* extract using Soxhlet extraction with 75% ethanol and reported that the MIC values antibacterial activity against *Escherichia coli* and *Staphylococcus aureus* (*S. aureus*) were 0.075 g/mL and 0.05 g/mL, respectively [36]. Thapa et al. reported that the chloroform leaf extract of *A. adenophora* exhibited the most pronounced activity against *E. coli* and *Proteus vulgaris*, also showed consistent antibacterial effects against *Klebsiella pneumoniae*, *Bacillus subtilis, Staphylococcus aureus*, and *Salmonella typhi*, all yielding inhibition zones of 8 mm [37]. In a subsequent study, Thapa et al. demonstrated that the essential oil of *A. adenophora* produced an inhibition zone of ~12 mm against *Staphylococcus aureus* and exerted inhibitory effects on *Klebsiella pneumoniae*, *Bacillus subtilis*, *Micrococcus luteus*, with inhibition zones of 8 mm, 7 mm, and 7 mm, respectively [38]. Liu et al. isolated 9-oxo-10,11-dehydroageraphorone (ODA) from *A. adenophora*, this compound showed potent antibacterial activity against *Ralstonia solanacearum*, with MICs ranging from 0.25 to 1 mg/mL and *M*BCs of 2 mg/mL for all tested strains [39]. Zhang et al. further isolated eight thymol derivatives from the roots of *A. adenophora*. Among these compounds, 7-formyl-9-isobutyryloxy-8-hydroxythymol exhibited potent antibacterial activity, with a minimum inhibitory concentration (MIC) of 3.9 μg/mL, supporting its potential as a natural lead compound for antibacterial drug development [26].

#### 4.1.1. Antibacterial Activity of Endophytic Microorganisms

In recent years, Ren et al. isolated an endophytic *Bacillus velezensis* (*B. velezensis*) strain Ea73 from *A. adenophora*. The fermentation broth exhibited broad-spectrum antibacterial activity against five pathogenic bacteria, namely *Escherichia coli*, *Salmonella typhimurium*, *Pseudomonas aeruginosa*, *Klebsiella pneumoniae*, and *S. aureus*, the inhibition zone against *S. aureus* reached 32.2 mm [10]. Two cyclic dipeptides, cyclo(*L*-Pro-*L*-Val) and cyclo(*L*-Leu-*L*-Pro), were further purified from the Ea73 fermentation broth. Both compounds exhibited moderate inhibitory activity against *S. aureus* and *E. coli* [10]. Tang et al. further demonstrated that the cell-free supernatant of *B. velezensis* EA73 had a minimum biofilm eradication concentration (MBEC) of 1.3 mg/mL against *S. aureus* biofilms. This antibiofilm effect was associated with the downregulation of biofilm-related genes (e.g., *icaA*, *icaD*, and *sarA*), accompanied by reduced levels of extracellular polysaccharides, extracellular proteins, and extracellular DNA (eDNA), providing a theoretical basis for developing endophyte-derived antimicrobial agents from *A. adenophora* [40].

#### 4.1.2. Antibacterial Enhancement by Nanomaterials

Dua et al. employed an aqueous leaf extract of *A. adenophora* as both a bioreducing agent and a stabilizer to synthesize silver nanoparticles (AgNPs) via a green route. Mechanistic analyses indicated in antibacterial assays that AgNPs adhere to the bacterial cell surface and disrupt membrane integrity, resulting in leakage of intracellular components, concurrently, they induce the generation of reactive oxygen species, increasing the DNA strand-break rate by ~40% [41].

**Table 2:** An overview of antibacterial activity of extracts of *A. adenophora* and related products.

Source Category	Key Active Component/Material	Target Microorganism(s)	MIC or MBC or ZOI or EC_50_	Reference
Crude extracts	70% ethanol extract	*Escherichia coli*	MIC = 0.075 g/mL, ZOI = 13.3 mm	[36]
		*Staphylococcus aureus*	MIC = 0.05 g/mL, ZOI = 14.2 mm	
	Chloroform extract	*Escherichia coli* ATCC 8739	ZOI = 12 mm	[37]
		*Proteus vulgaris* ATCC 6380	ZOI = 12 mm	
	*Essential oil*	*Staphylococcus aureus* KCTC 1916	ZOI = 12 mm	[38]
		*Klebsiella pneumoniae* KCTC 2242	ZOI = 8 mm	
		*Bacillus subtilis* KACC 17047	ZOI = 7 mm	
		*Micrococcus luteus* KACC 13377	ZOI = 7 mm	
	ODA	*Ralstonia solanacearum*	MIC = 0.25–1 mg/mL, MBC = 2 mg/mL	[11,39]
		*Fusarium oxysporum*	EC_50_ = 0.3 mg/mL	
	7-formyl-9-isobutyryloxy-8-hydroxythymol	*Staphylococcus aureus*, *Bacillus thuringiensis*	MIC = 7.8 μg/mL	[39,42]
		*Bacillus cereus*	MIC = 3.9 μg/mL	
		*Escherichia coli*, *Salmonella enterica*	MIC = 15.6 μg/mL	
	7-hydroxy-dehydrotremetone	*Colletotrichum gloeosporioides*	ZOI = 17.3 mm	[43]
		*Colletotrichum musae*	ZOI = 17.2 mm	
		*Rhizoctonia solani*	ZOI = 16.4 mm	
		*Fusarium oxysporum f.* sp. *niveum*	ZOI = 13.9 mm	
		*Alternaria alternata*	ZOI = 14.4 mm	
Endophyte-derived products	Fermentation broth of *Bacillus velezensis* Ea73	*Staphylococcus aureus*	ZOI = 32.2 mm	[10]
	Cyclo(*L*-Pro-*L*-Val)	*Staphylococcus aureus*	MIC = 256 μg/mL	
	Cyclo(*L*-Pro-*L*-Val)	*Escherichia coli*	MIC = 512 μg/mL	
	Ethyl acetate extract of endophytic fungus *Penicillium sclerotigenum* DCL06	*Methicillin-resistant Staphylococcus aureus*	MIC = 0.5–1 mg/mL, MBC = 1–2 mg/mL	[24]
	Ethyl acetate extract of endophytic fungus *Diaporthe kochmanii* DCL09	*Escherichia coli* (SMU1710)	MIC = 0.5–1 mg/mL, MBC = 1–2 mg/mL	
Nanomaterials	Silver nanoparticles (AgNPs)	*Staphylococcus aureus*	MIC = 64.5 μg/mL	[41]
	Silver nanoparticles (AgNPs)	*Escherichia coli*	MIC = 82.5 μg/mL	

Note: ZOI, zone of inhibition; MIC, minimum inhibitory concentration; MBC, minimum bactericidal concentration; ODA, 9-oxo-10,11-dehydroageraphorone; EC_50_, half maximal effective concentration; MBEC, minimum biofilm eradication concentration.

Overall, the reported antibacterial activity of *A. adenophora* varies markedly among different studies, depending on the type of preparation, target microorganism, and evaluation index. Crude extracts and essential oils generally showed inhibitory effects at relatively high concentrations, whereas some purified compounds, such as thymol derivatives, exhibited lower MIC values at the μg/mL level. Endophyte-derived products and green-synthesized nanomaterials also displayed antibacterial potential, but their activity and mechanisms differed from those of plant extracts and isolated phytochemicals. Therefore, the current evidence indicates that *A. adenophora* possesses antibacterial potential, but these findings are mainly based on in vitro assays and are not yet sufficient to support its direct use as a mature antimicrobial alternative in animal production. At present, *A. adenophora* is more appropriately regarded as a source of antibacterial lead compounds or bioactive materials that require further purification, standardization, safety evaluation, and in vivo validation.

### 4.2. Inhibitory Effects Against Fungi

Chaiwaree et al. reported that the ethanol extract of *A. adenophora* exhibited pronounced antidermatophytic activity, with MIC of 0.04 mg/mL against *Trichophyton mentagrophytes* and <0.0025 mg/mL against *Trichophyton rubrum* [11]. Liu et al. isolated cadinene-type sesquiterpenes from the methanolic extract of *A. adenophora*, their antifungal activities were evaluated for the first time against wood-decay fungi. At 100 μg/mL, cadinene-type sesquiterpenes showed the highest inhibition against *Inonotus cuticularis* M1308 strain *and Inonotus cuticularis* M1239 strain, reaching 59.7% and 44.4% [44]. Liu et al. reported that ODA isolated from *A. adenophora* had an EC50 of 0.325 mg/mL (48 h) against *Fusarium oxysporum* [39]. Zheng et al. further found that the benzofuran derivative 7-hydroxy-dehydrotremetone, isolated from the roots of *A. adenophora*, exhibited broad-spectrum inhibitory activity. At a dose of 50 mg per paper disc, the inhibition zone diameters against *Colletotrichum gloeosporioides*, *Colletotrichum musae*, *Rhizoctonia solani*, *Fusarium oxysporum f.* sp. *niveum*, and *Alternaria alternata* were 17.3 mm, 17.2 mm, 16.4 mm, 13.9 mm, and 14.4 mm, respectively [43]. These results suggest that plant-derived natural products with activity against multiple agricultural pathogenic fungi may offer useful leads for the development of green fungicides.

### 4.3. Antioxidant Activity

In in vitro evaluations of the antioxidant activity of *A. adenophora*, the 2,2-Diphenyl-1-picrylhydrazyl (DPPH) radical-scavenging assay is the most commonly used. The median inhibition concentration (IC_50_) values of its chloroform extract, 70% ethanol extract, ethyl acetate extract, petroleum ether extract, and aqueous extract were 1460 μg/mL, 74 μg/mL, 123 μg/mL, 232 μg/mL, and 55 μg/mL, respectively [37,45,46]. In the 2,2′-azino-bis (3-ethylbenzothiazoline-6-sulfonic acid) (ABTS) assay, the IC_50_ values of its aqueous extract, 95% ethanol extract, ethyl acetate extract, n-butanol extract, and petroleum ether extract were 13.2 μg/mL, 22.9 μg/mL, 29.7 μg/mL, 29.6 μg/mL, and 30.9 μg/mL, respectively [37,45,46]. Dua et al. synthesized AgNPs via a green approach using an aqueous leaf extract of *A. adenophora*, and the resulting nanoparticles exhibited a DPPH-scavenging IC_50_ of 49.0 μg/mL [41]. In a t-BHP-induced oxidative-injury model in Caco-2 cells, Li et al. reported that pretreatment with the aqueous extract at 1 and 5 mg/mL significantly increased the cell viabilities to 69.0%, 78.9% and reduced intracellular ROS levels of 43.9% and 82.8%, respectively [16]. Okyere et al. isolated and identified an endophytic *Bacillus toyonensis* strain (i.e., SAU-19) from *A. adenophora*. In a high-fat diet mouse model, SAU-19 significantly attenuated the elevation of malondialdehyde (MDA) in serum and intestinal tissues and effectively reversed the decline in key antioxidant indices, including superoxide dismutase (SOD), catalase (CAT), and glutathione (GSH), highlighting endophyte-derived resources as a promising direction for the development and utilization of the antioxidant potential of *A. adenophora* [47].

Additionally, the antioxidant potential of *A. adenophora* can also be interpreted from its phytochemical composition. As abovementioned, *A. adenophora* contains multiple flavonoids and phenolic acids, including quercetin, kaempferol, isorhamnetin, caffeic acid, and p-coumaric acid, as well as their glycosidic derivatives, which are all chemical classes commonly associated with redox regulation [9,30]. Flavonoids rich in phenolic hydroxyl groups can directly donate hydrogen atoms or electrons to neutralize reactive radicals, and can also enhance endogenous antioxidant defense systems [48]. For instance, quercetin can trigger Nrf2 nuclear translocation and elevate GSH to defend cells against oxidative stress, kaempferol and isorhamnetin mitigate oxidative damage partially via the Nrf2/HO-1 pathway [49,50,51]. In parallel, phenolic acids such as caffeic acid and p-coumaric acid exhibit well-documented radical-scavenging and anti-lipid-peroxidation activities [52]. Therefore, the antioxidant activity of *A. adenophora* extracts is mainly attributed to the flavonoids and phenolic acids.

### 4.4. Anti-Inflammatory Activity

Okyere et al. identified the endophytic strain *Bacillus toyonensis* SAU-19 from *A. adenophora* and conducted a 35-day oral gavage study in high-fat diet mice. Compared with the high-fat diet group, SAU-19 significantly reduced the production of the pro-inflammatory cytokines IL-1β and TNF-α in small intestinal tissue, while maintaining the mRNA and protein levels of the anti-inflammatory cytokines IL-4 and IL-10. In addition, SAU-19 decreased the abundance of pathogenic bacteria such as *Escherichia coli*, *Salmonella*, and *Staphylococcus* and increased beneficial taxa including *Lactobacillus* in the small intestine and cecum [47]. Shi et al. reported that isolated essential oil from *A. adenophora* at concentrations ≤20 mg/mL showed no cytotoxicity toward RAW264.7 macrophages and significantly suppressed lipopolysaccharide (LPS)-induced the expression of Toll-like receptor 4 (*TLR4*) and *IL-6* mRNAs, and upregulated the expression of *IL-10* mRNA. These results indicated that isolated essential oil from *A. adenophora* can inhibit the inflammatory response via blocking the TLR4 pathway [53].

### 4.5. Insecticidal and Acaricidal Activity

Sun et al. reported that an ethanolic extract of *A. adenophora* exhibited strong nematicidal activity against *Bursaphelenchus xylophilus*. The same study further demonstrated pronounced fumigant toxicity against adult stored-product insects, including *Sitophilus oryzae* and *Sitophilus zeamais* [14]. Li et al. found that an aqueous extract of *A. adenophora* exhibited pronounced molluscicidal activity across developmental stages of *Limax maximus* [54]. Mechanistic analyses indicated that the extract disrupted redox homeostasis and detoxification metabolism, as evidenced by decreased activities of antioxidant enzymes, including SOD, peroxidase, and CAT, and detoxification enzymes such as cytochrome P450 and glutathione S-transferase. Changes in acetylcholinesterase activity were also observed, suggesting that interference with neural and metabolic functions may contribute to its molluscicidal effects [54].

Rajeswary et al. reported that the methanolic leaf extract of *A. adenophora* exerted concentration-dependent larvicidal activity against *Culex quinquefasciatus*, *Aedes aegypti*, and *Anopheles stephensi* [55]. Samuel et al. further showed that petroleum ether and chloroform extracts displayed pronounced larvicidal activity against *C. quinquefasciatus* [12]. In addition, constituents such as safranal and dihydrocoumarin exhibited toxic effects against *Solenopsis invicta*, further supporting the potential of *A. adenophora*-derived compounds as sources of botanical insecticidal agents [9].

Collectively, these findings indicate that *A. adenophora* possesses insecticidal, nematicidal, and molluscicidal activities against several agriculturally and medically important pests. Its extracts and isolated constituents may therefore provide candidate materials for botanical pesticide development and integrated pest management. However, most available evidence is derived from laboratory bioassays. Further studies are required to establish standardized preparations, clarify target-specific mechanisms, assess environmental persistence and non-target toxicity, and verify efficacy under field conditions. 

### 4.6. Others

It was reported that *A. adenophora* exerts significant regulatory effects on mammalian intestinal and rumen microbial communities [56]. Okyere et al. isolated and identified two strains of *Bacillus toyonensis* (SAU-19 and SAU-20) from *A. adenophora*. Studies revealed that in a high-fat diet-induced dysbiosis mouse model, oral administration of SAU-19 and SAU-20 significantly reduced body weight gain, improved intestinal structural integrity, increased ileal villus height and crypt depth, and upregulated mRNA expression levels of tight junction proteins (i.e., occludin and ZO-1). This restored gut microbiota balance, reduced levels of pathogenic bacteria such as *Escherichia coli*, *Salmonella,* and *Staphylococcus aureus* in the ileal and cecal contents, while increasing the abundance of beneficial *Lactobacillus* spp. [47].

**Figure 1:**
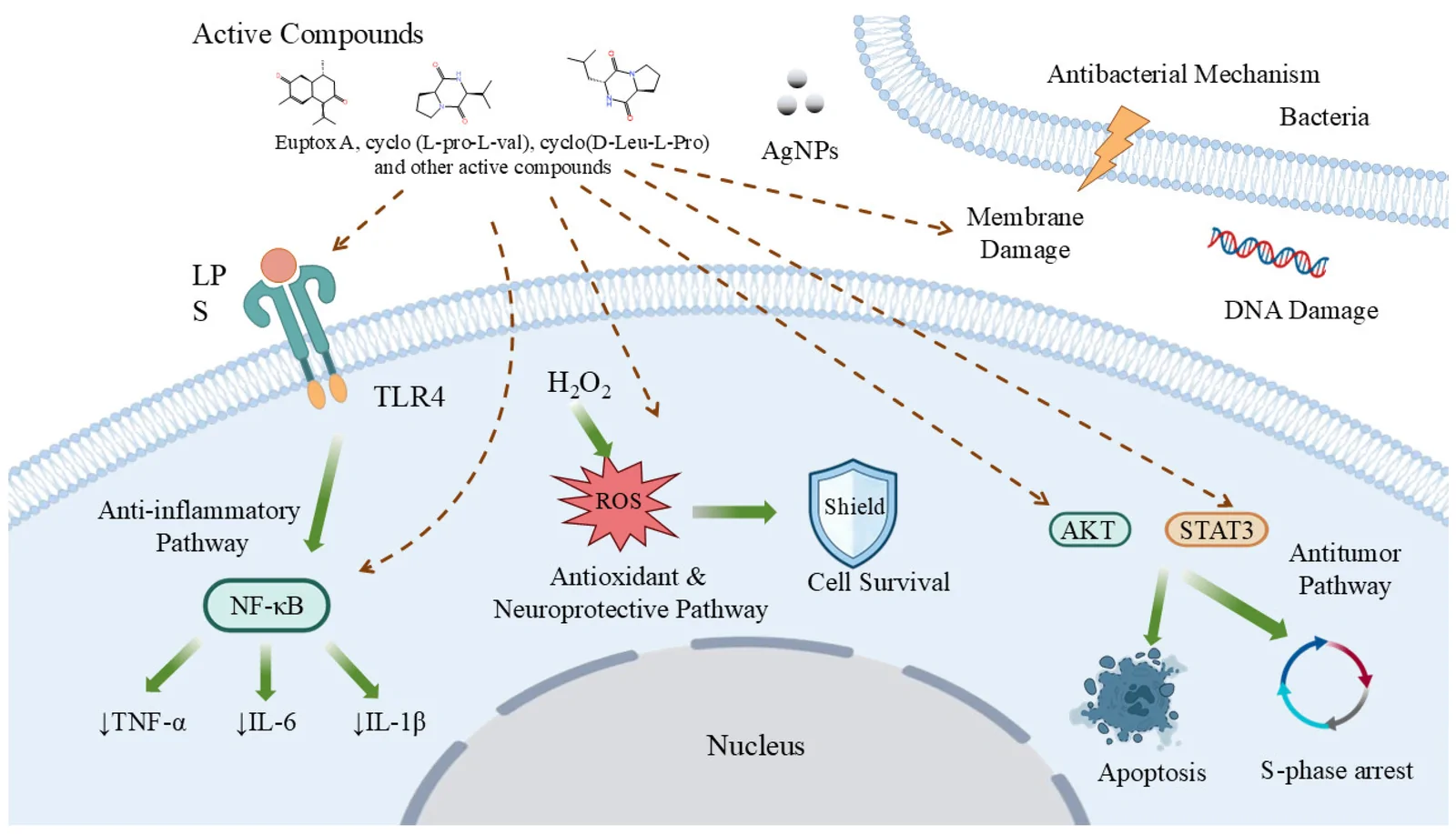
Comprehensive biological activities of *A. adenophora* containing bioactive compounds in mammalian cells.

In summary, *A. adenophora* contains a variety of bioactive constituents, including terpenoids, flavonoids, and polyphenols, which exhibit multiple biological activities, including antimicrobial, antioxidant, anti-inflammation, and gut health regulation effects. Notably, some components can scavenge ROS, alleviate oxidative stress, and suppress inflammation-related signaling pathways, which together contributed to the beneficial effects observed in mammalian cells (Figure 1). However, Current research has not conducted further comparative studies to determine which compound is primarily responsible for anti-inflammatory activity. Further research is still needed.

## 5. Toxicological Profiles of *A. adenophora*

Among the toxic constituents identified in *A. adenophora*, cadinene-type sesquiterpenes [e.g., 2-deoxo-2-(acetyloxy)-9-oxoageraphorone (DAOA), 9-oxoageraphorone (OA), and ODA] are prominent [57]. The following sections summarize toxic effects and mechanisms of *A. adenophora* in the intestine, liver, spleen, and rumen epithelium of animals, and are summarized in Table 3. Importantly, current evidence suggests that the toxic effects of *A. adenophora* are not uniform, but are strongly influenced by exposure dose and animal species. Therefore, the toxicity evaluation of *A. adenophora* should be interpreted from both dose–response and species-specific perspectives, especially when considering its potential utilization in animal production.

### 5.1. Hepatotoxicity

Ouyang et al. conducted acute and subacute toxicity assays in Kunming mice, thereby elucidating the hepatotoxicity of three cadinene-type sesquiterpenes from *A. adenophora*. In a single-dose oral acute toxicity test, the median lethal dose (LD_50_) of DAOA in male mice was 926 mg/kg, whereas the LD_50_ values of OA and ODA were both 1470 mg/kg, indicating that DAOA is more hepatotoxic than OA and ODA. In a 7-day oral gavage subacute study, DAOA treatment at 150 mg/kg significantly increased serum total bilirubin (TB) as well as alanine aminotransferase (ALT), aspartate aminotransferase (AST), and alkaline phosphatase (ALP) levels, whereas OA induced significant elevations in these liver-injury indices only at 300 mg/kg, supporting the view that these sesquiterpenes can induce dose-dependent cholestasis and liver injury and may exert immunotoxic effects [57]. These findings provide relatively direct evidence for dose-dependent hepatotoxicity, as lower doses of DAOA were sufficient to induce significant increases in liver injury biomarkers, whereas higher doses were required for OA to produce comparable effects. Shao et al. fed goats with 40% *A. adenophora* powder for 90 days. Marked inflammatory response, accompanied by focal steatosis, ballooning degeneration, hepatocellular swelling, and inflammatory cell infiltration, was detected in the liver tissue, it also significantly upregulated the expression of *IL-9*, *IL-17*, *TNF-α*, C-reactive protein (*CRP*), *IL-1β*, and *IL-6* mRNAs and significantly downregulated the expression of IL-4, IL-10, and TGF-β1 mRNAs. Untargeted metabolomics results showed that *A. adenophora* exposure also dysregulated hepatic energy and lipid metabolism [64]. In a 60-day feeding study conducted on rats, the subjects were provided with a diet consisting of 3:7 *A. adenophora* powder and standard feed, Ren et al. noted that DAOA, OA, and ODA identified from *A. adenophora* have been established as key toxic constituents responsible for animal liver injury, providing additional evidence for the hepatotoxic risk associated with this plant [62]. Euptox A is a major hepatotoxic component of *A. adenophora*, and it can induce hepatic lesions such as focal hepatocellular necrosis and degenerative desquamation of bile duct epithelium and cell cycle arrest and cell apoptosis in hepatocyte of experimental animals [65]. Kaushal et al. reported that hepatotoxic constituents of *A. adenophora* can be extracted with methanol and partially purified, following 48 h of exposure in rats. Serum total bilirubin, conjugated bilirubin, ALP, 5′-nucleotidase, ALT, and AST levels were markedly elevated. The histopathological alterations included bile duct dilatation and hyperplasia, periductal hepatocellular necrosis, and mononuclear cell infiltration. The toxic principles were mainly terpenoid compounds, which can induce cholestasis and hepatic parenchymal injury in rats [60].

Network toxicology predictions indicate that the primary toxic components of *A. adenophora* are 2-deoxy-2-acetoxy-9-oxo-cinnamaldehyde, 10Hβ-9-oxo-cinnamaldehyde, 10Hα-9-oxo-cinnamaldehyde, nerolidol, and 9-oxo-10,11-dehydro-phellandrene, all of which comply with Lipinski’s rules and exhibit good oral bioavailability [29]. A comprehensive metabolomic analysis was conducted on sheep liver tissue samples that had been fed a diet containing *A. adenophora* for a period of 90 days. The analysis revealed a significant enrichment of metabolites associated with histidine metabolism, arachidonic acid metabolism, and neuroactive ligand-receptor interactions. Transcriptomic analysis further identified epidermal growth factor receptor (*EGFR*), G protein-coupled bile acid receptor 1 (*GPBAR1*), and peroxisome proliferator-activated receptor alpha (*PPARα*) identified as core genes [29].

**Figure 2:**
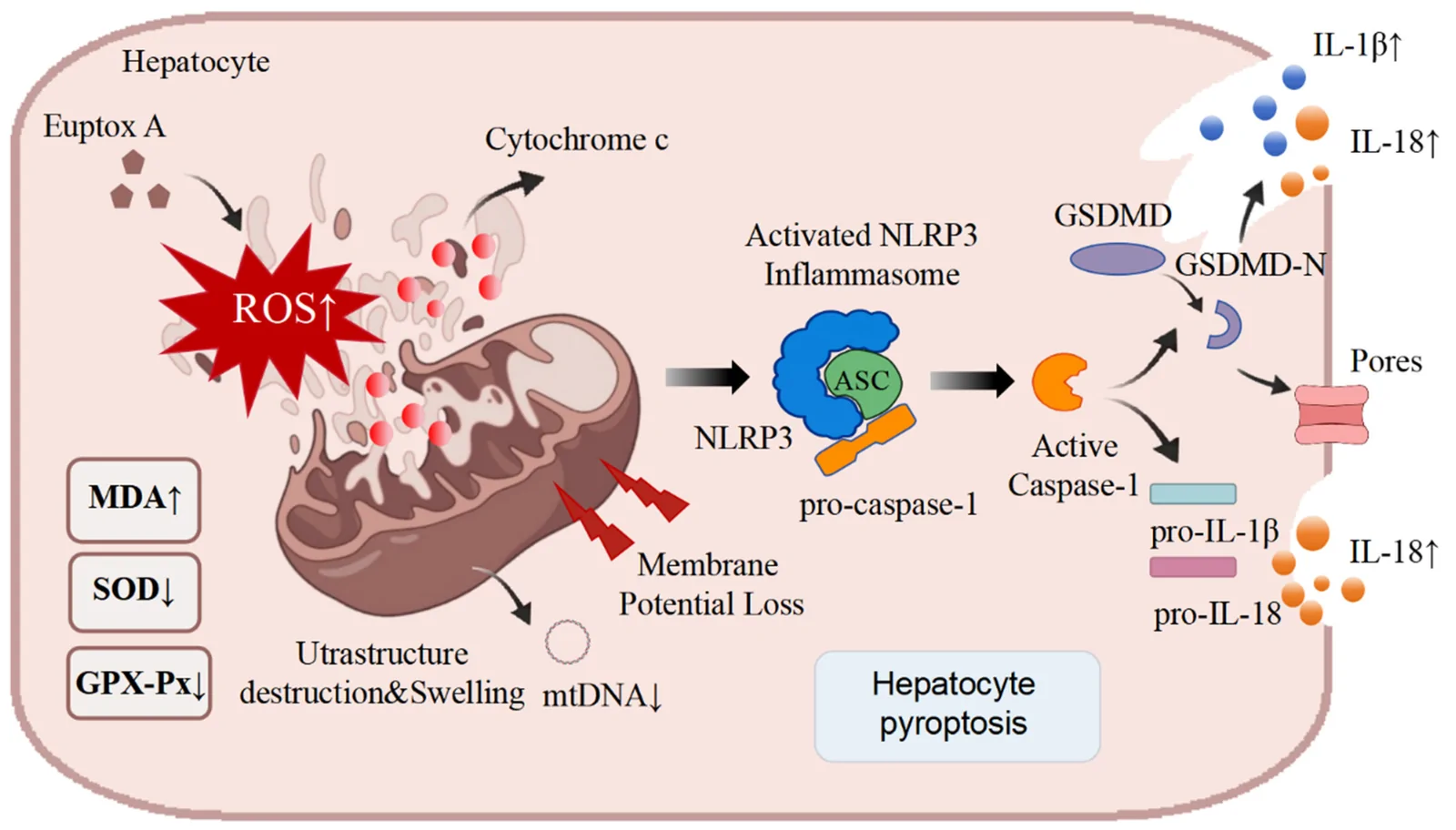
Mechanism of *A. adenophora*-induced hepatotoxicity via ROS-mitochondrial axis drives NLRP3-mediated pyroptosis.

After 6 weeks of feeding mice a diet containing 300 g/kg *A. adenophora*, a marked increase in ROS levels, and MDA contents and decreases in the activities of SOD and GSH-Px were detected in the liver tissues [66]. Concurrently, mitochondrial ultrastructure was disrupted with increased swelling, while Na^+^K^+^-ATPase and Ca^2+^Mg^2+^-ATPase activities and mitochondrial DNA copy number were markedly decreased [66]. Furthermore, it has been demonstrated that this process results in the upregulation of NOD-like receptor family pyrin domain containing 3 (NLRP3) inflammasome expression, which subsequently leads to elevated levels of caspase-1 activity and the secretion of IL-1β and IL-18. Ultimately, this culminates in the occurrence of hepatocyte pyroptosis [66]. This evidence indicates that *A. adenophora*-induced liver injury involves the oxidative stress, mitochondrial, pyroptosis pathways. The detailed mechanism of *A. adenophora*-induced liver injury presented in Figure 2.

### 5.2. Gastrointestinal Toxicity

After 60 days of feeding rats with a *A. adenophora* powder and basal diet in a 3:7 ratio, the small intestine exhibited villus shedding, inflammatory cell infiltration, and hemorrhage. The duodenal villus height decreased, the crypt depth increased, and the villus height/crypt depth (V/C) ratio significantly decreased. Concurrently, serum intestinal injury markers diamine oxidase and D-lactic acid were significantly increased and the expressions of several intestinal tight junction proteins, including occludin, claudin-1, and ZO-1 mRNA were significantly downregulated [58]. Similarly, Wang et al. observed marked pathological intestinal damage in goats fed 40% *A. adenophora* powder for 90 days, with significantly more severe injury in the small intestine than in the large intestine [59].

The primary toxic mechanism involves the activation of the myosin light-chain kinase (MLCK) and Rho-associated coiled-coil-containing protein kinase (ROCK) signaling pathway. It has been reported that euptox A has the capacity to induce the upregulation of MLCK and ROCK1/2 genes and protein expressions in ileal tissue of goats [59]. Concurrently, MLCK mediates myosin light chain (MLC2) phosphorylation, while ROCK inhibits myosin light chain phosphatase (MLCP) activity by phosphorylating myosin phosphatase target subunit 1 (MYPT1). Together, these processes induce centripetal contraction of the cytoskeleton, which ultimately leading to increased intestinal permeability [59].

Additionally, it was also reported that *A. adenophora* can specifically damage the ruminal epithelium of goats. After feeding a diet containing 40% powdered plant material for 90 days, the epithelium of both the dorsal and ventral rumen sacs became darker (with black–green adherent deposits), and ruminal papillae were sparse. The mRNA and protein expression levels of the tight-junction proteins occludin, claudin-1, and ZO-1 in epithelial cells decreased by 30% to 50% [67].

### 5.3. Spleen Toxicity

Ren et al. conducted an in vivo study in 7-week-old male rats fed a diet containing *A. adenophora* powder for 60 days. It significantly increased the spleen index and marked histopathology injury (i.e., white pulp atrophy and congestion/hemorrhage of the red pulp) in the spleen tissues of rats were observed [62]. *A. adenophora* exposure can also disrupt the splenic fibroblastic reticular cell (FRC) network, which was evidenced by a disruption of the Th1/Th2 immune balance in rats [62,68]. He et al. performed a 3-month feeding experiment in Saanen dairy goats receiving diets containing 40%, 60%, or 80% *A. adenophora*, respectively. They found that *A. adenophora* induced dose-dependent apoptosis in goat splenocytes [63]. In a 7-day subacute toxicity study, Ouyang et al. orally administered the cadinane-type sesquiterpene DAOA (at 300 mg/kg body weight) derived from *A. adenophora*, to Kunming mice. It significantly increased the spleen index and was accompanied by thymic atrophy, demonstrating a direct toxic effect of *A. adenophora* sesquiterpenes on immune organs such as the spleen. This phenomenon has been linked to the process of splenocyte apoptosis [57]. Mechanistically, this pro-apoptotic effect depended on the mitochondrial pathway, characterized by upregulation of Bax and downregulation of Bcl-2, release of cytochrome c (Cyt c) from mitochondria into the cytosol, and activation of the caspase-9/caspase-3 apoptotic cascade. In addition, *A. adenophora* exposure caused G0/G1 cell-cycle arrest in splenocytes, which ultimately resulted in splenocyte apoptosis and impaired splenic immune function [63].

Collectively, the above findings suggest that the toxicological effects of *A. adenophora* are jointly governed by exposure dose, duration of intoxication, and species-specific susceptibility. The severity of damage not only progressively increases with higher exposure levels and longer intoxication periods, but also differs markedly across species in terms of target organs and overall sensitivity. Ouyang et al. and Sun et al. showed that sesquiterpene toxins induced dose-dependent oxidative stress and pathological injury in the liver and spleen of mice, as evidenced by progressively increased ROS and MDA levels as well as aggravated histopathological lesions at higher doses [57,68]. Liao et al. and He et al. further demonstrated that *A. adenophora* extracts and euptox A significantly increased apoptosis and cellular injury in HeLa cells and goat splenocytes in a concentration-dependent manner; at higher concentrations, substantial apoptosis was induced through the mitochondrial and caspase-dependent pathways, whereas little or no obvious toxicity was observed at lower concentrations [13,63]. In an in vivo goat study [63,69], Wang et al. found that intestinal barrier injury progressively worsened with prolonged exposure to *A. adenophora* (30–60 d), and serum DAO and D-lactic acid levels increased in a time-dependent manner [67]. With regard to species-related variation, available studies suggest that horses are particularly susceptible to pulmonary toxicity, whereas cattle may exhibit anorexia, cessation of rumination, and photosensitization. In contrast, mice and rats mainly develop hepatotoxicity and cholestasis, while goats may display relatively different tolerance or altered feeding behavior depending on exposure level and plant source. Notably, even in rodent studies, toxic outcomes vary according to the geographic origin of the plant material and the method of preparation, suggesting that apparent species-related differences may also interact with the phytochemical heterogeneity of *A. adenophora*. 

Accumulating evidence suggests that different livestock species exhibit significant differences in susceptibility to non-detoxified *A. adenophora*, while the safe dose thresholds have not yet been systematically established for most species. Current evidence predominantly demonstrates that goats develop significant hepatic and rumen damage after long-term feeding of a diet containing 40% *A. adenophora* meal [64], and no clear safe feeding window has been identified for this group to date. In rodent studies, dietary administration of *A. adenophora* at doses ≥100 g/kg diet in mice resulted in dose-dependent exacerbation of hepatic inflammatory injury, oxidative stress and mitochondrial damage [68]. For purified cadinene-type sesquiterpenes, the 7-day subacute safe dose in mice was determined to be 75 mg/kg body weight [57]. 

Therefore, from the perspective of practical application, subsequent studies have gradually shifted from solely evaluating the toxicity of the raw plant to the research and development of detoxification strategies and detoxified products, with the aim of reducing sesquiterpene-related toxicity while retaining its valuable bioactive components for potential development in feed or veterinary medicine.

## 6. *A. adenophora* as a Functional Feed Additive and Its Detoxification

*A. adenophora* exhibits enormous biomass and well-balanced crude protein and amino acid profiles, conferring it high potential as an unconventional feed resource. The core toxic constituents of *A. adenophora* are cadinene-type sesquiterpenoids, among which euptox A is the key mediator of hepatotoxicity, pulmonary toxicity and immunotoxicity in livestock and poultry [70,71]. Secondary metabolites including tannins, coumarins and essential oils can also reduce feed palatability and exert synergistic toxic effects [72]. Therefore, all existing detoxification technology systems are centered on the degradation or removal of sesquiterpenoid toxins, while maximally preserving the nutritional and functional active components of the plant such as crude protein, amino acids and flavonoids, which eliminates the core barriers to its feed resource utilization [72]. This section systematically sorts out the principles and research advances of mainstream detoxification technologies for *A. adenophora*, and comprehensively evaluates the feeding efficacy of detoxified *A. adenophora* in pigs, broilers, laboratory animals and ruminants in terms of growth performance, product quality and multi-faceted toxicological safety, offering theoretical basis and practical reference for its further development as functional feed.

### 6.1. Various Detoxification Technologies

Among various detoxification technologies, microbial fermentation detoxification is the core technology with the most in-depth research and mature application at present, and also the cornerstone for realizing the large-scale feed resource utilization of *A. adenophora* [1,72]. Early studies have screened fungal strains capable of efficiently degrading the toxic components of *A. adenophora*. Among them, *Penicillium chrysogenum* and *Aspergillus ustus* achieved a tannin degradation rate of over 70.0%, a coumarin degradation rate of 67.0%, and a total essential oil degradation rate of 91.2%, without damaging the original nutritional components of the plant during fermentation. This work was the first to confirm the feasibility of microbial detoxification for feed production [72]. Subsequent studies further screened functional bacterial strains capable of specifically degrading euptox A from *A. adenophora* plants, contaminated soil, and the rumen of migratory goats, including *Klebsiella* sp., *Stenotrophomonas* sp., and *Pseudomonas* sp. [70,71]. Among them, the *Klebsiella pneumoniae* XC-08 strain isolated from goat rumen achieved a 94.3% degradation rate of 45.0 mg/L euptox A within 24 h [71]. Furthermore, strains such as *Klebsiella variicola* PLP G-17 LC and *Klebsiella pneumoniae* PLP G-17 SC can simultaneously degrade hydrolyzable tannins and euptox A in feed, providing core bacterial resources for the development of ruminant-specific detoxification bacterial inoculants [70]. The aerobic fermentation technology with composite bacterial strains optimized for practical production scenarios has also been systematically verified. After treatment with composite bacteria, the crude fiber content of *A. adenophora* decreased from 27.8% to 20.2%, while the crude protein content was stably maintained above 22.0%. While achieving complete degradation of toxins, this technology also optimized the fiber structure and nutritional ratio of the feed, enabling it to replace conventional energy feeds such as wheat bran, which lays a solid foundation for its industrial production [73]. In addition to microbial fermentation, detoxification technology based on the targeted enrichment and purification of active components provides a new avenue for the development of functional feed additives derived from *A. adenophora* [74]. Through processes including macroporous resin enrichment and recrystallization, chlorogenic acid products with varying purities can be prepared from the leaves of *A. adenophora*. While directionally enriching anti-inflammatory and antioxidant active components such as chlorogenic acid and flavonoids, this process almost completely eliminates sesquiterpenoid toxins [74]. In addition, processes including chemical pretreatment and ensiling have been verified to auxiliary enhance detoxification efficiency, and are compatible with the feed production requirements of varying scales [75].

Consistently, detoxified *A. adenophora* exhibited high safety as a feed additive. For example, Xie et al. used Kunming mice to evaluate the subchronic toxicity of detoxified *A. adenophora* powder. In this study, detoxified *A. adenophora* powder was added to the basal diet at 2%, 5%, and 10%, and the mice were continuously fed for 90 days [76]. The results showed that throughout the experimental period, the behavior, coat condition, fecal characteristics, water intake, feed intake, and body weight of mice in all treatment groups remained normal, and no mortality was observed. Serum biochemical indicators measured at 30, 60, and 90 days, including AST, ALT, BUN, total protein, and albumin, showed no significant differences from the control group. After 90 days, body weight in the 2%, 5%, and 10% detoxified *A. adenophora* powder groups continued to increase normally over time. Organ coefficients of the spleen, kidney, and liver showed no obvious differences from those of the control group, and histopathological sections of the liver, kidney, and spleen showed no abnormal microscopic lesions [76]. Li et al. used a combined toxicological evaluation scheme consisting of the Ames test, mouse bone marrow polychromatic erythrocyte micronucleus test, and chromosome aberration test of primary spermatocytes to evaluate the mutagenicity of biologically fermented detoxified *A. adenophora* powder. The results were all negative, with no dose–response relationship observed, indicating no mutagenic effect [77]. Liu et al. reported that detoxified *A. adenophora* powder had no obvious effects on feed intake, activity, body weight gain, implantation number, live fetuses, dead fetuses, or resorbed fetuses in pregnant mice. Fetal body weight, body length, and tail length showed no obvious differences from those of the negative control group, and no malformations were observed in external appearance, skeletal structure, or internal organs, indicated that detoxified *A. adenophora* powder had no maternal or embryotoxic effects [78]. Li et al. incorporated biologically fermented detoxified *A. adenophora* powder into the diet at doses of 10 g/kg and 20 g/kg and continuously fed New Zealand rabbits for 90 days [79]. The results showed that rabbits in all groups remained in good general condition throughout the experimental period, with no mortality, feed refusal, or abnormal behavior observed. During the trial, behavior, activity, feeding, and defecation were normal in all groups, and body weight gain, daily feed intake, and feed utilization showed no statistically significant differences compared with the control group [79]. Hematological parameters, serum biochemical indicators, organ coefficients, and histopathological examination showed that continuous feeding with 10 g/kg and 20 g/kg detoxified *A. adenophora* powder for 90 days caused no obvious adverse effects in rabbits [79]. The study also reported that the oral LD_50_ of fermented detoxified *A. adenophora* powder in rats, mice, and rabbits was greater than 10,000 mg/kg, indicating that it could be classified as practically non-toxic [79].

### 6.2. Application Effects and Safety Evaluation of Detoxified A. Adenophora as Feed Additive in Animals

#### 6.2.1. Swine

Yang et al. replaced wheat bran in swine diets with detoxified *A. adenophora* at proportions of 10%, 15%, and 20%, respectively, and formulated compound feeds for a 120-day feeding trial [73]. The results showed that pigs remained healthy throughout the experimental period, with no disease or adverse reactions observed [73]. In terms of production performance, the average daily gains of the control group and the 10%, 15%, and 20% detoxified *A. adenophora* groups were 558, 546, 527, and 508 g/head, respectively, all reaching the general domestic level of approximately 500 g/day, with no significant differences between the experimental groups and the control group [73]. The feed-to-gain ratios of the control group and the 10%, 15%, and 20% detoxified *A. adenophora* groups were 3.99:1, 4.05:1, 4.11:1, and 4.30:1, respectively, indicating an increasing trend in feed-to-gain ratio as the inclusion level increased [73]. Economic analysis showed that the cost per kilogram of weight gain and gross profit per pig in the 10% and 15% detoxified *A. adenophora* groups were not markedly different from those in the control group, whereas gross profit per pig decreased markedly when the inclusion level reached 20%. Therefore, the authors suggested that 15% may be a suitable inclusion level of detoxified *A. adenophora* in swine diets [73].

Song et al. further evaluated the effects of dietary detoxified *A. adenophora* powder on pork quality. In this study, Landrace crossbred pigs with body weights of 30–40 kg were fed complete diets supplemented with 10%, 15%, or 20% detoxified *A. adenophora* powder for 90 consecutive days, after which loin muscle samples were collected for analysis [80]. The results showed that replacement of part of the wheat bran with 10–20% detoxified *A. adenophora* powder did not adversely affect pork appearance, tissue condition, odor, taste, or boiled broth characteristics, all of which met the requirements of the NY/T843–2009 Green Food-Meat and Meat Products standard for fresh livestock meat [80]. In terms of physicochemical indicators, both moisture content and total volatile basic nitrogen were below the standard limits. With regard to hygienic indicators, inorganic arsenic, mercury, copper, lead, cadmium, chromium, tetracycline, oxytetracycline, chlortetracycline, chloramphenicol, and furazolidone were either not detected or were within the permitted limits. Microbiological indicators showed that the total bacterial count was less than 10 CFU/g, coliforms were less than 300 CFU/kg, and neither Salmonella nor diarrheagenic Escherichia coli was detected [80].

#### 6.2.2. Broilers

Broilers are relatively sensitive to feed safety and can therefore be used to verify the safety and feasibility of the detoxification process for *A. adenophora*. Zhang et al. used broilers as experimental animals and supplemented diets with up to 6% detoxified *A. adenophora* powder in an 8-week feeding trial [75]. The results showed that the survival rates of the experimental groups ranged from 93.3% to 96.7%, with no obvious difference from the control group. The weight gain rate and feed-to-meat ratio of broilers were within the normal range, and no growth inhibition was observed [75]. These findings indicate that, under nutritionally comparable diet formulations, the addition of detoxified *A. adenophora* feed did not adversely affect broiler growth [75]. After slaughter, the appearance and tissue morphology of the heart, liver, spleen, lung, kidney, and other organs were normal, the meat was tender and free from abnormal odor, and the heavy metal contents in the liver met food hygiene standards.

#### 6.2.3. Ruminants

Ruminants, particularly goats, are important target animals for evaluating the feed utilization potential of *A. adenophora*, because they are commonly exposed to this plant in invaded grazing areas. Direct feeding of non-detoxified *A. adenophora* to goats may cause damage to hepatocytes, renal tissues, myocardium, and lymphoid tissues, and may also affect immune function. In contrast, detoxified *A. adenophora* not only showed no obvious toxicity but also exhibited improved palatability [81]. Zhou et al. evaluated *A. adenophora* as a goat feed by incorporating detoxified *A. adenophora* into compound diets at 20%, 30%, and 40%, and compared these treatments with a non-detoxified group [82]. The results showed that feed intake in the 20%, 30%, and 40% detoxified *A. adenophora* groups increased by 31.7%, 30.2%, and 14.3%, respectively, with the 20% inclusion level showing better performance. In terms of weight gain, the 20% detoxified *A. adenophora* group showed a 136.8% increase in average weight gain, while the 30% and 40% groups also showed certain growth-promoting effects. In contrast, the non-detoxified *A. adenophora* group showed a negative weight-gain trend [82].

In summary, the feed utilization of *A. adenophora* has currently formed a detoxification technology system with microbial fermentation as the core and multiple technologies in synergy [72]. Multiple animal feeding trials and systematic toxicological assessments have verified that detoxified *A. adenophora* at appropriate inclusion levels causes no acute toxicity, subchronic toxicity or genotoxicity in pigs, broilers, rabbits, goats and other animals, nor does it exert negative impacts on animal growth and development, organ function or meat quality. It can effectively replace conventional energy feeds such as wheat bran, with both remarkable ecological benefits and economic value [77,78,79,81,83,84]. Nevertheless, existing studies are still focused on basic safety validation and growth performance observation; the regulatory effects and mechanisms of detoxified *A. adenophora* on animal gut microbiota structure, immune function, antioxidant status and pathogen resistance remain poorly understood. Future research should further carry out systematic investigations focusing on the optimization of appropriate inclusion ratios, long-term feeding safety, elucidation of functional mechanisms and product quality improvement, so as to establish standardized feeding application specifications and advance the industrial application process of *A. adenophora* feed resource utilization [78].

### 6.3. Advantages and Limitations of A. adenophora as a Feed Additive

*A. adenophora* has multiple unique advantages as a feed additive. First, as an invasive weed, *A. adenophora* features tremendous biomass and extensive distribution in southwestern China. Its raw material cost is much lower than that of artificially cultivated medicinal plants, making its feed products cost-competitive in the market [72]. Second, *A. adenophora* contains a wide variety of active components, including functional substances such as flavonoids, phenolic acids and polysaccharides, which exert multiple biological functions including antioxidant, anti-inflammatory, antibacterial and immunomodulatory activities [72,78]. Third, developing *A. adenophora* into a feed additive has certain ecological value: it can not only alleviate the ecological damage caused by its invasion, but also create economic value, realizing the resource utilization of harmful organisms [84]. Fourth, *A. adenophora* possesses a unique antiparasitic function, which is rare among conventional plant-derived feed additives and provides it with distinctive application value in the prevention and control of parasitic diseases [85].

However, *A. adenophora* also has obvious limitations when used as a feed additive. The sesquiterpenoid toxins in *A. adenophora*, especially euptox A, are the fundamental constraint on its direct feed utilization [72,85]. The LD_50_ of euptox A in mice is 1470 mg/kg body weight [57]. Undetoxified *A. adenophora* (i.e., including euptox A) at high dose level can cause damage to the liver, lungs, intestinal tract and immune system in animals [67,71]. The main toxic pathogenesis of *A. adenophora* is oxidative stress and inflammatory response, which can induce hepatotoxicity, splenotoxicity and other related diseases in animals [70,71]. Therefore, all utilization schemes must take prior detoxification as a prerequisite, which increases the process complexity and production cost [78,86]. Furthermore, the detoxification process is not yet fully mature. Current research on *A. adenophora* detoxification is still in the developmental stage. Systematic studies are still needed in aspects including strain screening for microbial fermentation detoxification, process parameter optimization and large-scale production, and there is still no unified standardized evaluation system for the efficiency and safety of different detoxification methods [77]. Third, the application evidence is not sufficiently systematic. Compared to conventional additives such as astragalus polysaccharides and allicin, which have abundant long-term feeding and industrial application data, existing feeding studies on *A. adenophora* mainly focus on short-term safety evaluation and small-scale trials, lacking large-scale, multi-center and long-term production performance data [3,9]. Fourth, there are large species-specific differences. Different animal species show significantly different sensitivity to *A. adenophora* toxins. In goats, feeding diets containing undetoxified *A. adenophora* significantly upregulates the mRNA expression of inflammatory cytokines including *TNF-α*, *IFN-γ*, *IL-1β*, *IL-2*, *IL-6* and *IL-10* in the ruminal epithelium, and downregulates the expression of tight junction proteins such as occludin and claudin-1 [59]. Poultry are highly sensitive to the toxins, while ruminants have relatively higher tolerance due to the detoxification effect of rumen microorganisms [76]. Such species differences require the development of differentiated detoxification standards and inclusion level schemes for different animals, which further increases the complexity of application and popularization.

## 7. Conclusions and Prospects

*A. adenophora* should be regarded as a chemically rich but intrinsically hazardous biomass rather than as a ready-to-use feed additive. Its terpenoids, flavonoids, phenolic acids, and endophyte-derived metabolites exhibit a broad range of reported biological activities, particularly antimicrobial, antioxidant, anti-inflammatory, and gut-regulatory effects. However, most of these effects have been demonstrated in chemical assays, cell models, or small-scale animal experiments. More importantly, the same plant contains cadinene-type sesquiterpenes and other constituents capable of inducing hepatobiliary injury, intestinal and ruminal barrier disruption, splenic immune dysfunction, oxidative stress, inflammation, and programmed cell death. Therefore, the presence of potentially beneficial activities cannot, by itself, justify the direct inclusion of raw or insufficiently characterized *A. adenophora* in animal diets.

Based on the available evidence, the most feasible feed-related application is not the untreated plant, but a standardized and adequately detoxified feed ingredient produced through validated microbial fermentation, selective extraction, or fractionation. Such products may retain useful nutrients and selected antioxidant or antimicrobial constituents while reducing the concentrations of euptox A and other toxic sesquiterpenes. Nevertheless, current feeding studies demonstrate preliminary feasibility rather than commercial readiness. Many studies were conducted on a limited number of animals, used relatively short exposure periods, or lacked standardized characterization of the starting material, detoxification efficiency, and residual toxin concentrations. Species-specific safe inclusion levels also remain uncertain, and findings obtained in rodents or individual livestock species cannot be directly generalized across animal-production systems.

Purified phytochemicals and endophyte-derived products may represent more controllable application routes than whole-plant preparations because their composition, potency, and toxicological profiles can be characterized more precisely. Some isolated thymol derivatives and microbial metabolites may provide leads for antimicrobial development, whereas the insecticidal, nematocidal, and molluscicidal properties of *A. adenophora* may support separate applications in botanical pest control. These pesticidal effects, however, should be interpreted primarily as evidence of biological potency and possible toxicity rather than as support for feed use. Their development will require independent evaluation of mammalian toxicity, environmental persistence, non-target effects, and field efficacy.

Future research should move beyond descriptive screening of biological activity toward a risk-controlled product-development framework. Priorities include: (i) characterization of geographic, seasonal, and plant-part variation in both beneficial and toxic constituents; (ii) establishment of validated marker compounds and quantitative limits for residual sesquiterpene toxins; (iii) standardization and scale-up of detoxification processes, including batch-to-batch quality control and stability assessment; (iv) long-term, dose–response feeding studies in target livestock species that assess growth performance, feed efficiency, organ pathology, immune and reproductive outcomes, product quality, and tissue residues; and (v) direct comparison with established feed additives to determine whether detoxified *A. adenophora* provides meaningful biological or economic advantages. The environmental consequences of harvesting, processing, and transporting this invasive plant should also be evaluated to avoid unintentionally facilitating its further spread.

In conclusion, the practical value of *A. adenophora* lies not in the direct use of the invasive plant, but in its controlled conversion into chemically defined, detoxified, and application-specific products. Until reproducible detoxification standards, species-specific safety thresholds, and long-term efficacy data are established, its use in animal nutrition should remain cautious and experimental. A safety-first strategy integrating phytochemical standardization, toxicological validation, and production-scale assessment is essential for determining whether this invasive and toxic plant can be transformed into a genuinely useful feed or veterinary resource.

## Figures and Tables

**Table 1:** A representative part of the isolated chemical constituents from *A. adenophora*.

No.	Compound	Molecular Formula	Structure	Reference
Terpenoids				
1	9-Oxo-10,11-dehydroageraphorone (named euptox A)	C_15_H_20_O_2_	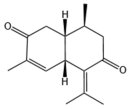	[28]
2	9-Oxo-ageraphorone	C_15_H_22_O_2_	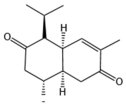	[28]
3	9β-Hydroxyageraphorone	C_15_H_24_O_2_	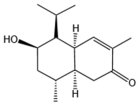	[28]
4	β-Bisabolene	C_15_H_24_	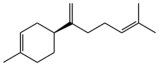	[23]
5	p-cymene	C_10_H_14_	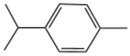	[23]
6	Bornyl acetate	C_12_H_20_O_2_	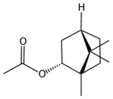	[23]
7	γ-Curcumene	C_15_H_24_	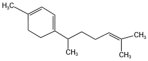	[23]
8	β-Caryophyllene	C_15_H_24_	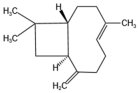	[23]
9	Epi-α-cadinol	C_15_H_26_O_2_	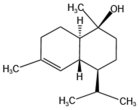	[23]
10	Geraniol	C_10_H_18_O	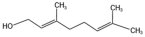	[23]
11	Germacrene D	C_15_H_24_	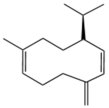	[23]
12	β-Farnesene	C_15_H_24_	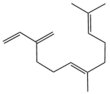	[23]
13	Nerolidol	C_15_H_26_O	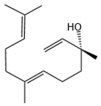	[23]
14	2-Deoxy-2-(acetyloxy)-9-oxoageraphorone (named DAOA)	C_17_H_22_O_4_	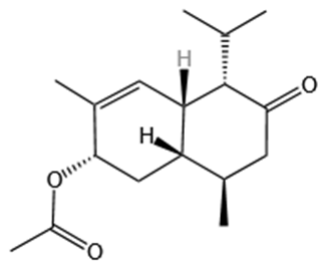	[29]
15	Amorpha-4,7(11)-diene	C_15_H_24_	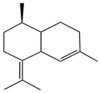	[28]
16	(–)-Amorph-4-en-7-ol	C_15_H_26_O	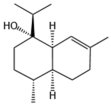	[28]
17	α-Phellandrene	C_10_H_16_	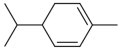	[23]
18	δ-2-Carene	C_10_H_16_	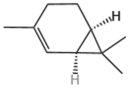	[23]
19	Safranal	C_10_H_14_O	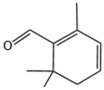	[9]
Flavonoids				
20	Isorhamnetin	C_16_H_12_O_7_	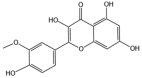	[9]
21	Quercetin	C_15_H_10_O_7_	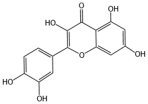	[30]
22	Genistein	C_15_H_10_O_5_	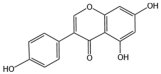	[9]
23	Quercetin-3-O-β-D-glucoside	C_21_H_20_O_12_	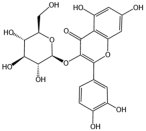	[31]
24	Kaempferol-3-O-β-D-glucopyranoside	C_21_H_20_O_11_	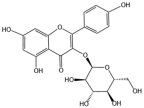	[31]
25	Fortunellin	C_28_H_32_O_14_	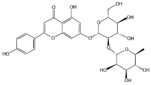	[31]
26	Kaempferol	C_15_H_10_O_6_	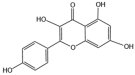	[31]
Phenolic acids				
27	Caffeic acid	C_9_H_8_O_4_	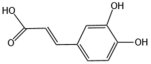	[9]
28	3-Coumaric acid	C_9_H_8_O_3_	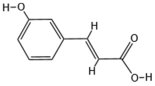	[9]
29	2,3-Dihydroxybenzoic acid	C_7_H_6_O_4_	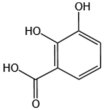	[9]
30	Salicylic acid	C_7_H_6_O_3_	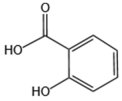	[9]
Cyclic dipeptides				
31	Cyclo (*L*-Pro-*L*-Val)	C_10_H_16_N_2_O_2_	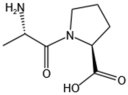	[10]
32	Cyclo (*L*-Leu-*L*-Pro)	C_11_H_18_N_2_O_2_	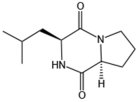	[10]
33	Cyclo (*L*-Phe-*L*-Pro)	C_14_H_16_N_2_O_2_	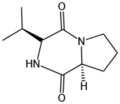	[10]
Other Active Components				
34	Phenyllactic acid	C_9_H_10_O_3_	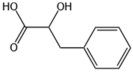	[9]
35	Citric acid	C_6_H_8_O_7_	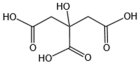	[9]
36	Glutaconic acid	C_5_H_6_O_4_	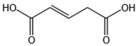	[9]
37	Dihydrocoumarin	C_9_H_8_O_2_	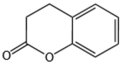	[9]

**Table 3:** A summary of toxic effects and mechanisms of *A. adenophora* on major target organs in animals.

Toxicity Category	Animal Model	Key Toxic Constituents	Pathological Characteristics	Mechanisms of Action	References
Intestinal toxicity	Sprague–Dawley rats	Euptox A	Villus desquamation, inflammatory-cell infiltration, mucosal hemorrhage, decreased villus height, and reduced barrier integrity.	Activation of MLCK/ROCK signaling and downregulation of occludin, claudin-1, and ZO-1, following to increase intestinal permeability.	[58]
	Black goats	Euptox A and sesquiterpene derivatives	Necrosis and sloughing of intestinal epithelial cells, lamina propria edema, ileal villus disruption, and crypt hyperplasia.	MLCK/ROCK activation and membrane damage by sesquiterpene derivatives, then impair intestinal epithelial integrity.	[59]
Hepatotoxicity	Rats	Methanolic leaf extract of *A. adenophora* and a partially purified terpenoid toxic fraction	Elevated bilirubin, ALP, 5′-nucleotidase, ALT, and AST; bile-duct dilatation/hyperplasia; periductal hepatocellular necrosis.	Selective injury to the bile duct epithelium leads to cholestasis and secondary hepatocellular necrosis.	[60]
	Kunming mice	DAOA, OA, ODA	Disrupted hepatic lobules, focal/bridging necrosis, nuclear pyknosis, fibrosis, and increased ALT, AST, and ALP; DAOA showed the lowest LD_50_.	The exposure to high doses can cause cholestasis and hepatocellular necrosis through biliary and hepatocellular injury. Concurrent damage to immune organs (thymus and spleen) may then amplify hepatic inflammation.	[57]
	Chengdu Ma goats	2-deoxo-2-acetoxy-9-oxoageraphorone and nerolidol	Focal steatosis, ballooning degeneration, hepatocellular swelling, inflammatory infiltration, and increased ALT, AST, ALP, GGT, TC and total bilirubin (TBiL).	Imbalance of hepatic cytokine networks, i.e., pro-inflammatory mediators (*TNF-α*, *IL-1β*, *IL-6*, *IL-9*, *IL-17*, and *CRP*) were significantly upregulated at the mRNA level, whereas anti-inflammatory mediators (*IL-4*, *IL-10*, and *TGF-β1*) were significantly downregulated. Disruption of hepatic metabolic homeostasis, with perturbations in neuroactive ligand–receptor interaction and pyrimidine metabolism pathways.	[61]
Splenic toxicity	Rats	No specific single compound identified	Increased spleen index, white-pulp atrophy, red-pulp congestion/hemorrhage, and impaired splenic immune architecture.	Disruption of the splenic FRC network with downregulation of chemokines *CCL21* and *CCL19*, resulting in reduced homing of CD3+ T cells to the spleen, concomitant skewing of the splenic Th1/Th2 balance toward a Th1 phenotype, ultimately suppressing splenic immune function and causing immune injury.	[62]
Splenic toxicity	Goats	No specific single compound identified	Dose-dependently increases apoptosis in splenocyte. Caspase-9 and caspase-3 activity and expression were significantly increased, Bax protein was upregulated and Bcl-2 was downregulated. Typical splenic lesions included splenic corpuscle atrophy and disordered lymphocyte arrangement.	Activation of the mitochondrial apoptotic pathway: upregulation of Bax and downregulation of Bcl-2, leading to collapse of mitochondrial membrane potential and cytochrome c release, thereby triggering the caspase-9/caspase-3 cascade, induction of G0/G1 cell-cycle arrest in splenocytes.	[63]

Note: ALP, alkaline phosphatase; ALT, alanine aminotransferase; AST, aspartate aminotransferase; LD_50_, median lethal dose; GGT, gamma-glutamyl transferase; TBiL, total bilirubin; TC, total cholesterol; *CRP*, C-reactive protein; *FRC*, fibroblastic reticular cell; *CCL19*, C-C motif chemokine ligand 19; *CCL21*, C-C motif chemokine ligand 21; Th1/Th2, T helper 1/T helper 2.

## Data Availability

No new data were created or analyzed in this study. Data sharing is not applicable to this article.

## References

[1] Yao M., Liu L., Hao Z., Shen J., Dai C. (2026). A narrative review of *Ginkgo biloba* extract: Biological function, molecular mechanisms, and applications in animal production. Antioxidants.

[2] Ferrara F., Castagna T., Pantolini B., Campanardi M.C., Roperti M., Grotto A., Fattori M., Dal Maso L., Carrara F., Zambarbieri G. (2024). The challenge of antimicrobial resistance (AMR): Current status and future prospects. Naunyn-Schmiedeberg’s Arch. Pharmacol..

[3] Chattopadhyay M.K. (2014). Use of antibiotics as feed additives: A burning question. Front. Microbiol..

[4] Staff A. (2006). European Union Bans All Antibiotic Growth Promoters.

[5] Ivanova S., Sukhikh S., Popov A., Shishko O., Nikonov I., Kapitonova E., Krol O., Larina V., Noskova S., Babich O. (2024). Medicinal plants: A source of phytobiotics for the feed additives. J. Agric. Food Res..

[6] Du E., Li P., Chen Y., Lin H., Xu Q., Huang X., Gui F. (2025). Positive feedback effect of rhizosphere *Bacillus* on the growth and defense of *Ageratina adenophora*. BMC Plant Biol..

[7] Das M., Acharya B., Saquib M., Chettri M. (2018). Effect of aqueous extract and compost of invasive weed *Ageratina adenophora* on seed germination and seedling growth of some crops and weeds. J. Biodivers. Conserv. Bioresour. Manag..

[8] Ruan Y., Gao X., Hussain M., Qin X., Ning J., Yang D., Zhu T., Qin D., Ye M., Wu G. (2025). Methyl Indole-3-Acetate (MEIAA) mediated stem curvature and apical meristem necrosis in *Ageratina adenophora*: Impacts on cell wall components, vascular system integrity, and key metabolic pathways. BMC Plant Biol..

[9] Wu M., Luo R., Hussain M., Wu W., Li S., Guo Z., Jia B., Bi G., Gao X., Wu G. (2025). Nature-identical safranal and dihydrocoumarin from *Ageratina adenophora* ((Spreng., 1970) King and H. Rob.) target energy metabolism to control *Solenopsis invicta* Buren, 1972 (Hymenoptera: Formicidae). Insects.

[10] Ren Z., Xie L., Okyere S.K., Wen J., Ran Y., Nong X., Hu Y. (2022). Antibacterial activity of two metabolites isolated from endophytic bacteria *Bacillus velezensis* Ea73 in *Ageratina adenophora*. Front. Microbiol..

[11] Chaiwaree S., Pongpaibul Y., Thammasit P. (2022). Anti-dermatophyte activity of the aqueous extracts of Thai medicinal plants. Braz. J. Biol..

[12] Samuel L., Lalrotluanga, Muthukumaran R.B., Gurusubramanian G., Senthilkumar N. (2014). Larvicidal activity of *Ipomoea cairica* (L.) Sweet and *Ageratina adenophora* (Spreng.) King & H. Rob. plant extracts against arboviral and filarial vector, *Culex quinquefasciatus* Say (Diptera: Culicidae). Exp. Parasitol..

[13] Fu J., Hu L., Shi Z., Sun W., Yue D., Wang Y., Ma X., Ren Z., Zuo Z., Peng G. (2021). Two metabolites isolated from endophytic fungus *Coniochaeta* sp. F-8 in *Ageratina adenophora* exhibit antioxidative activity and cytotoxicity. Nat. Prod. Res..

[14] Sun W., Liu S.S., Zhao C.C. (2023). Biological properties of active compounds from *Ageratina adenophora*. SAGE Open Med..

[15] Liao F., He J., Wen Y., Tang Z., Yang F., Zhi R., Qiao Y., Chen B., Hu Y. (2025). *Ageratina adenophora* essential oil: A promising miticidal agent against *Sarcoptes scabiei*, and its effects on key enzymatic pathways. Biol. Control.

[16] Li Z.Q., Ni J., Zhu X.Y., Zhang C.Z., An R., Yang X., She R., Yang X.Y. (2024). Antioxidant and anti-inflammatory function of *Eupatorium adenophora* Spreng leaves (EASL) on human intestinal Caco-2 cells treated with tert-butyl hydroperoxide. Sci. Rep..

[17] Liu F., Du L., Li T., Liu B., Guo J., Zhang G., Zhang Y., Liu W., Pan Y., Zhang Y. (2025). Chromosome-level genome assembly of the crofton weed (*Ageratina adenophora*). Sci. Data.

[18] Dwivedi S., Baunthiyal M. (2025). Assessing the genetic diversity of *Eupatorium adenophorum* (*Ageratina adenophora*) through the development of ISSR-derived SCAR (sequence characterized amplified region) markers. Fitoterapia.

[19] Mi L., Liu H., Zhang J., Guo Y., Shi J., Lu Y., Cheng J., Wang H., Cheng D., Valverde B.E. (2025). Low-temperature-induced singlet oxygen adaptation decreases susceptibility to the mycotoxin TeA in invasive plant *Ageratina adenophora*. Plant Physiol. Biochem..

[20] Zhang K., Gu R., Yang Y., Yan J., Ma Y., Shen Y. (2025). Recent distribution changes of invasive Asteraceae species in China: A five-year analysis (2016–2020). J. Environ. Manag..

[21] Darji T.B., Adhikari B., Pathak S., Neupane S., Thapa L.B., Bhatt T.D., Pant R.R., Pant G., Pal K.B., Bishwakarma K. (2021). Phytotoxic effects of invasive *Ageratina adenophora* on two native subtropical shrubs in Nepal. Sci. Rep..

[22] Wu T., Duan Q., Zhang H., Xiu W., Jiang Z. (2025). Differences in soil nutrient and microbial characteristics between invasive *Ageratina adenophora* and native plant communities. PLoS ONE.

[23] Palá-Paúl J., Pérez-Alonso M.J., Velasco-Negueruela A., Sanz J. (2002). Analysis by gas chromatography-mass spectrometry of the volatile components of *Ageratina adenophora* Spreng., growing in the Canary Islands. J. Chromatogr. A.

[24] Wen J., Okyere S.K., Wang J., Huang R., Wang Y., Liu L., Nong X., Hu Y. (2023). Endophytic fungi isolated from *Ageratina adenophora* exhibits potential antimicrobial activity against multidrug-resistant *Staphylococcus aureus*. Plants.

[25] Yin B., Li X., Li Z.X., Zhu X.X., Zhang L., Zhou X.L., Xu J.B., Chen F.Z., Tang P., Gao F. (2023). Adenophorone, an unprecedented sesquiterpene from *Eupatorium adenophorum*: Structural elucidation, bioinspired total synthesis and neuroprotective activity evaluation. Angew. Chem. Int. Ed. Engl..

[26] Zhang M., Ouyang J.-K., Xu Q.-L., Liu S.-B., Qian T., Dong L.-M., Tan J.-W. (2021). Thymol derivatives with antibacterial and cytotoxic activity from the aerial parts of *Ageratina adenophora*. RSC Adv..

[27] Chanu K.D., Sharma N., Kshetrimayum V., Chaudhary S.K., Ghosh S., Haldar P.K., Mukherjee P.K. (2023). *Ageratina adenophora* (Spreng.) King & H. Rob. Standardized leaf extract as an antidiabetic agent for type 2 diabetes: An in vitro and in vivo evaluation. Front. Pharmacol..

[28] Liu Y., Luo S.H., Hua J., Li D.S., Ling Y., Luo Q., Li S.H. (2021). Characterization of defensive cadinenes and a novel sesquiterpene synthase responsible for their biosynthesis from the invasive *Eupatorium adenophorum*. New Phytol..

[29] Huang R., Okyere S.K., Shao C., Yousif M., Liao F., Wang X., Wen J., Wang J., Hu Y. (2023). Hepatotoxicity effects of *Ageratina adenophora*, as indicated by network toxicology combined with metabolomics and transcriptomics. Ecotoxicol. Environ. Saf..

[30] Chanu K.D., Thoithoisana S., Kar A., Mukherjee P.K., Radhakrishnanand P., Parmar K., Sharma N. (2024). Phytochemically analysed extract of *Ageratina adenophora* (Sprengel) R.M.King & H. Rob. initiates caspase 3-dependant apoptosis in colorectal cancer cell: A synergistic approach with chemotherapeutic drugs. J. Ethnopharmacol..

[31] Li M., Gao X., Lan M., Liao X., Su F., Fan L., Zhao Y., Hao X., Wu G., Ding X. (2020). Inhibitory activities of flavonoids from *Eupatorium adenophorum* against acetylcholinesterase. Pestic. Biochem. Physiol..

[32] Geng H. (2024). Chemical constituents and their bioactivities of plants from the genus *Eupatorium* (2015–present). Biology.

[33] Nong X., Li S.H., Chen F.Z., Wang J.H., Xie Y., Fang C.L., Liu T.F., He R., Gu X.B., Peng X.R. (2014). Isolation and identification of acaricidal compounds in *Eupatorium adenophorum* petroleum ether extract and determination of their acaricidal activity against *Psoroptes cuniculi*. Vet. Parasitol..

[34] Liu Y., Yang T., He Y., Zuo A., Li Q., Nie S., Hu S., Yan X. (2025). 9β-hydroxy-ageraphoron enhances TMV resistance in tobacco by activating the SA signaling pathway through upregulation of MYB and DOF genes. J. Agric. Food Chem..

[35] Ren Z., Okyere S.K., Xie L., Wen J., Wang J., Chen Z., Ni X., Deng J., Hu Y. (2022). Oral administration of *Bacillus toyonensis* strain SAU-20 improves insulin resistance and ameliorates hepatic steatosis in type 2 diabetic mice. Front. Immunol..

[36] Zhang X.M., Zhou Y.Z., Zhang Y.F. (2020). Antibacterial activity of *Ageratina adenophora* against *Escherichia coli* and *Staphylococcus aureus*. Guangdong J. Anim. Vet. Sci..

[37] Thapa A., Pokharel A., Karki H., Yadav R., Paudel N., Bharati S., Shrestha T., Maharjan B., Karanjit S., Shrestha R. (2022). Phytochemical analysis, cytotoxicity, antibacterial and antioxidant activities of extracts of leaf of *Ageratina adenophora* (Spreng.). Amrit Res. J..

[38] Thapa A., Maharjan B., Bharati S., Shrestha T., Homagai P., Bhattarai D., Shrestha R. (2024). Chemical composition, antibacterial, antioxidant, and cytotoxicity activities of essential oil of leaf of *Ageratina adenophora* (Spreng.) R.M. King and H. Rob. J. Nepal Chem. Soc..

[39] Liu X., Ouyang C., Wang Q., Li Y., Yan D., Yang D., Guo M., Cao A. (2016). Evaluation of antibacterial and antifungal properties of 9-oxo-10,11-dehydroageraphorone extracted from *Eupatorium adenophorum*. J. Plant Dis. Prot..

[40] Tang Z., Yousif M., Okyere S.K., Liao F., Peng S., Cheng L., Yang F., Wang Y., Hu Y. (2025). Anti-biofilm properties of cell-free supernatant from *Bacillus velezensis* EA73 by in vitro study with *Staphylococcus aureus*. Microorganisms.

[41] Dua T.K., Giri S., Nandi G., Sahu R., Shaw T.K., Paul P. (2023). Green synthesis of silver nanoparticles using *Eupatorium adenophorum* leaf extract: Characterizations, antioxidant, antibacterial and photocatalytic activities. Chem. Zvesti.

[42] Zhang M., Wang H., Hussain M., Qi J., Ma C., Lan M., Gao X., Wu G. (2020). Identification and functional assessment of endophytic bacterial diversity in *Ageratina adenophora* (Sprengel) and their interactions with the host plant. S. Afr. J. Bot..

[43] Zheng G., Luo S., Li S., Hua J., Li W., Li S. (2018). Specialized metabolites from *Ageratina adenophora* and their inhibitory activities against pathogenic fungi. Phytochemistry.

[44] Liu Y., Wang D., Hu S., Wu J., Luo J., Cao M., Yan X. (2023). Antifungal activity of cadinane-type sesquiterpenes from *Eupatorium adenophorum* against wood-decaying fungi. Chem. Biodivers..

[45] Giri S., Sahu R., Paul P., Nandi G., Dua T.K. (2022). An updated review on *Eupatorium adenophorum* Spreng. [*Ageratina adenophora* (Spreng.)]: Traditional uses, phytochemistry, pharmacological activities and toxicity. Pharmacol. Res. Mod. Chin. Med..

[46] Zhang C.Z., Yuan C.L., Cheng Y.T., She R., Xiao W. (2022). Antioxidant activity of the invasive alien species *Ageratina adenophora*. Chem. Bioeng..

[47] Okyere S.K., Wen J., Cui Y., Xie L., Gao P., Zhang M., Wang J., Wang S., Ran Y., Ren Z. (2022). *Bacillus toyonensis* SAU-19 and SAU-20 isolated from *Ageratina adenophora* alleviates the intestinal structure and integrity damage associated with gut dysbiosis in mice fed high fat diet. Front. Microbiol..

[48] Faysal M., Dehbia Z., Zehravi M., Sweilam S.H., Haque M.A., Kumar K.P., Chakole R.D., Shelke S.P., Sirikonda S., Nafady M.H. (2024). Flavonoids as potential therapeutics against neurodegenerative disorders: Unlocking the prospects. Neurochem. Res..

[49] Arredondo F., Echeverry C., Abin-Carriquiry J.A., Blasina F., Antúnez K., Jones D.P., Go Y.-M., Liang Y.-L., Dajas F. (2010). After cellular internalization, quercetin causes Nrf2 nuclear translocation, increases glutathione levels, and prevents neuronal death against an oxidative insult. Free Radic. Biol. Med..

[50] Yao H., Sun J., Wei J., Zhang X., Chen B., Lin Y. (2020). Kaempferol protects blood vessels from damage induced by oxidative stress and inflammation in association with the Nrf2/HO-1 signaling pathway. Front. Pharmacol..

[51] Yang J.H., Shin B.Y., Han J.Y., Kim M.G., Wi J.E., Kim Y.W., Cho I.J., Kim S.C., Shin S.M., Ki S.H. (2014). Isorhamnetin protects against oxidative stress by activating Nrf2 and inducing the expression of its target genes. Toxicol. Appl. Pharmacol..

[52] Genaro-Mattos T.C., Maurício Â.Q., Rettori D., Alonso A., Hermes-Lima M. (2015). Antioxidant activity of caffeic acid against iron-induced free radical generation-a chemical approach. PLoS ONE.

[53] Shi Z. (2019). The chemically isolated fractions of *Ageratina adenophora* essential oil inhibit lipopolysaccharide-induced inflammatory response in RAW264.7 cells. Chin. J. Cell. Mol. Immunol..

[54] Li H., Zhao R., Pan Y., Tian H., Chen W. (2024). Insecticidal activity of *Ageratina adenophora* (Asteraceae) extract against *Limax maximus* (Mollusca, Limacidae) at different developmental stages and its chemical constituent analysis. PLoS ONE.

[55] Rajeswary M., Govindarajan M. (2013). Mosquito larvicidal and phytochemical properties of *Ageratina adenophora* (Asteraceae) against three important mosquitoes. J. Vector Borne Dis..

[56] Fuke N., Nagata N., Suganuma H., Ota T. (2019). Regulation of gut microbiota and metabolic endotoxemia with dietary factors. Nutrients.

[57] Ouyang C.B., Liu X.M., Liu Q., Bai J., Li H.Y., Li Y., Wang Q.X., Yan D.D., Mao L.G., Cao A. (2014). Toxicity assessment of cadinene sesquiterpenes from *Eupatorium adenophorum* in mice. Nat. Prod. Bioprospect..

[58] Cui Y., Okyere S.K., Gao P., Wen J., Cao S., Wang Y., Deng J., Hu Y. (2021). *Ageratina adenophora* disrupts the intestinal structure and immune barrier integrity in rats. Toxins.

[59] Wang J., Wang S., Okyere S.K., Wen J., Wang X., Huang R., Tang Z., Cao S., Deng J., Ren Z. (2024). *Ageratina adenophora* causes intestinal integrity damage in goats via the activation of the MLCK/ROCK signaling pathway. Toxicon.

[60] Kaushal V., Dawra R.K., Sharma O.P., Kurade N.P. (2001). Hepatotoxicity in rat induced by partially purified toxins from *Eupatorium adenophorum* (*Ageratina adenophora*). Toxicon.

[61] Shao C., Huang R., Okyere S.K., Muhammad Y., Wang S., Wang J., Wang X., Hu Y. (2024). Study on the chronic inflammatory injury caused by *Ageratina adenophora* on goat liver using metabolomics. Toxicon.

[62] Ren Z., Gao P., Okyere S.K., Cui Y., Wen J., Jing B., Deng J., Hu Y. (2021). *Ageratina adenophora* inhibits spleen immune function in rats via the loss of the FRC network and Th1–Th2 cell ratio elevation. Toxins.

[63] He Y., Mo Q., Hu Y., Chen W., Luo B., Wu L., Qiao Y., Xu R., Zhou Y., Zuo Z. (2015). *E. adenophorum* induces cell cycle arrest and apoptosis of splenocytes through the mitochondrial pathway and caspase activation in saanen goats. Sci. Rep..

[64] Shao C.Y. (2024). Effects of *Ageratina adenophora* Poisoning on Liver Metabolism and Inflammatory Factors in Goats. Master’s Thesis.

[65] Okyere S.K., Mo Q., Pei G., Ren Z., Deng J., Hu Y. (2020). Euptox A Induces G0/GI arrest and apoptosis of hepatocyte via ROS, mitochondrial dysfunction and caspases-dependent pathways in vivo. J. Toxicol. Sci..

[66] Sun W. (2020). Study on Oxidative Stress, Mitochondrial Damage and Pyroptosis in Mouse Liver Induced by *Ageratina adenophora*. Ph.D. Dissertation.

[67] Wang X., Wang J., Okyere S.K., Huang R., Shao C., Yousif M., Deng J., Hu Y. (2024). *Ageratina adenophora* damages the rumen epithelium via inducing the expression of inflammatory factors in goats. J. Anim. Sci..

[68] Sun W., Zeng C., Yue D., Liu S., Ren Z., Zuo Z., Deng J., Peng G., Hu Y. (2019). *Ageratina adenophora* causes spleen toxicity by inducing oxidative stress and pyroptosis in mice. R. Soc. Open Sci..

[69] Liao F., Hu Y., Wu L., Tan H., Luo B., He Y., Qiao Y., Mo Q., Wang Y., Zuo Z. (2015). Induction and mechanism of HeLa cell apoptosis by 9-oxo-10, 11-dehydroageraphorone from *Eupatorium adenophorum*. Oncol. Rep..

[70] Sharma D., Mal G., Kannan A., Bhar R., Sharma R., Singh B. (2017). Degradation of euptox A by tannase-producing rumen bacteria from migratory goats. J. Appl. Microbiol..

[71] Liao F., Hu Y., Tan H., Wu L., Wang Y., Huang Y., Mo Q., Wei Y. (2014). Acaricidal activity of 9-oxo-10,11-dehydroageraphorone extracted from *Eupatorium adenophorum* in vitro. Exp. Parasitol..

[72] Han L., Tang H., Guo Y.J. (2010). Research progress of *Ageratina adenophora* as feed raw material. Jiangxi Feed.

[73] Yang F.G., Duan J.J., Zhu G.L. (1998). Study on detoxified *Ageratina adenophora* as feed raw material for swine. Grain Feed Ind..

[74] Liu B., Dong B., Yuan X., Guo Y., Zhang L., Zhao B. (2017). Simultaneous detoxification and preparative separation of chlorogenic acid from *Eupatorium adenophorum* by combined column chromatography. Sep. Sci. Technol..

[75] Zhang W.D., Jiang Y.H., Zhao F., Yu X.H. (1992). Study on feeding broilers with detoxified *Ageratina adenophora*, a harmful subtropical invasive weed. China Herbiv. Sci..

[76] Xie Q.X., Zhang J.M., Zhang W. (2013). Research progress on the invasive species *Ageratina adenophora*. Anim. Husb. Feed Sci..

[77] Li H.Y., Wang R., Zhang Z.L., Xu M., Wang R., Xiu H.D. (2012). Toxicological study of detoxified *Ageratina adenophora* meal. Chin. Occup. Med..

[78] Liu H., Song Z.H., Feng J. (2012). Research progress on detoxified feed of *Ageratina adenophora*. Heilongjiang J. Anim. Husb. Vet. Med..

[79] Li H.Y., Wang R., Zhang Z.L., Xu M., Ouyang H. (2010). 90-day subchronic toxicity evaluation of *Ageratina adenophora* meal in rabbits. J. Toxicol..

[80] Song Z.H., Liu H. (2013). Effects of dietary supplementation with detoxified *Ageratina adenophora* meal on pork quality. Heilongjiang J. Anim. Husb. Vet. Med..

[81] Zhou Z.W., Duan X.H., Xu C., Kui J.X. (2007). Study on *Ageratina adenophora* used as ruminant feed. Grassl. Anim. Husb..

[82] Zhou Z.W., Duan X.H., Xu C., Kui J.X. (2006). Experimental study on feeding goats with *Ageratina adenophora*. Feed Ind..

[83] Li H.Y., Ouyang H., Wang R., Zhang Z.L., Xu M. A 90-day feeding experiment of rabbits with *Ageratina adenophora* meal as a new feed resource. Proceedings of the Academic Conference on Food, Feed Safety and Risk Assessment.

[84] Wu L.F. (2008). Pig breeding using detoxified *Ageratina adenophora* in Yun County. Yunnan Daily.

[85] Tang X.P., Duan G., Li X.H., Jiang H.C., Xiang X., Chang H. (2019). Biological characteristics of *Ageratina adenophora* and its application in animal husbandry production. Prog. Vet. Med..

[86] Li C., Li H.Y., Zhang Z.L., Xiu H.D., Gu J.S. (2015). Study on immunotoxicity of *Ageratina adenophora* extracts in mice. J. Toxicol..

